# Rapid Infusion Rituximab for Maintenance Therapy: Is It Feasible?

**DOI:** 10.1155/2013/629283

**Published:** 2013-10-31

**Authors:** Jolly Patel, Melissa Ho, Viet Ho, Celeste Bello, Benjamin Djulbegovic, Lubomir Sokol, Gene Wetzstein

**Affiliations:** ^1^Department of Pharmacy, H. Lee Moffitt Cancer Center, 12902 Magnolia Drive Tampa, FL 33612, USA; ^2^Department of Malignant Hematology, H. Lee Moffitt Cancer Center, 12902 Magnolia Drive Tampa, FL 33612, USA

## Abstract

Rituximab is an anti-CD-20 monoclonal antibody used in the management of lymphoproliferative disorders. The use of maintenance rituximab has improved progression free survival and overall survival in follicular lymphomas. Although rapid rituximab infusions have been studied extensively, there is little data on the use of rapid infusions during maintenance therapy for low grade lymphomas. The primary objective of this retrospective analysis was to evaluate the incidence of Grade 3 and 4 toxicities with maintenance rapid infusion rituximab according to the Common Terminology Criteria for Adverse Events version 4 (CTC v. 4). Secondary objectives included evaluating all grade infusion related adverse events and correlation of adverse events with varying schedules of rituximab maintenance therapy. All patients who received rapid infusion rituximab as maintenance therapy for low grade lymphoma between December 2007 and November 2011 were included. Rapid rituximab infusions were administered over 90 minutes. Demographic, laboratory and clinical data were collected. A total of 109 patients received 647 rapid rituximab infusions. Three patients experienced an adverse reaction which resulted in one grade 1 infusion reaction and three grade 3 infusion reactions. No patients required hospitalization. All 3 patients received pharmacological and/or supportive care to relieve symptoms associated with the reaction.

## 1. Introduction

Rituximab (Rituxan) is a chimeric IgG1 monoclonal antibody that targets the CD20 receptor and is used in the management of lymphoproliferative disorders. Rituximab is FDA approved in the treatment of chronic lymphocytic leukemia (CLL), non-Hodgkin's lymphoma (NHL), and rheumatoid arthritis (RA). Rituximab is generally well tolerated; however, there are risks associated with infusion-related toxicity including hypersensitivity reactions such as fever, urticaria, hypotension, and cardiovascular and respiratory compromise [1]. The mechanism by which rituximab causes an infusion-related reaction is unclear but is thought to be due to the release of inflammatory cytokines [1]. The likelihood of infusion-related reactions is the highest with the first infusion and decreases with subsequent infusions with time to onset ranging from 30 to 120 minutes [2]. Other risk factors for developing an infusion-related reaction include large tumor burden, pulmonary infiltrates, elderly patients, and individuals diagnosed with chronic lymphocytic leukemia or mantle cell lymphoma [1]. Infusion related reactions typically resolve with supportive care and/or a decrease in the infusion rate. The overall incidence of infusion-related reactions varies based on diagnosis but ranges from 32 to 77% with the first infusion of rituximab [2]. The incidence of grade 3 or 4 infusion-related reactions is reported to be 9% for the first infusion of rituximab with cyclophosphamide, doxorubicin, vincristine, and prednisone (CHOP) or cyclophosphamide, vincristine, and prednisone (CVP) chemotherapy regimens [1].

In an effort to prevent infusion-related reactions, the manufacturer recommends that the first infusion of rituximab be given at a slow initial rate and gradually titrated upward which may take up to 6 to 8 hours [2]. Specifically the initial rituximab infusion should be started at a rate of 50 mg/hr, and if tolerated further titrate the rate by 50 mg/hr every 30 minutes to a maximum rate of 400 mg/hr. For subsequent infusions, the starting rate should be 100 mg/hr, and if tolerated, the rate can be further increased to 100 mg/hr every 30 minutes to maximum of 400 mg/hr. Additionally it is recommended to premedicate with acetaminophen and an antihistamine prior to each rituximab dose. Subsequent infusions may be given at a faster rate, but they may take up to 4 hours to infuse. Longer infusions result in a labor-intensive and time-consuming process [1]. 

Rituximab therapy in the maintenance setting has shown to improve progression-free survival and overall survival [3]. Maintenance rituximab can be administered according to several different schedules including one dose every 2 months, one dose every 3 months, or once weekly for 4 doses every 6 months [4, 5]. All schedules of maintenance therapy are continued for a duration of two years.

Studies have been conducted to determine the safety and feasibility of administering rapid rituximab; however, these studies did not include patients receiving rituximab maintenance for low-grade non-Hodgkin's lymphoma [3, 6–8]. Herein, we present our experience at H. Lee Moffitt Cancer Center with rapid infusion rituximab administered as maintenance therapy for low-grade non-Hodgkin's lymphoma. 

## 2. Methods

H. Lee Moffitt Cancer Center has been utilizing a rapid rituximab infusion protocol since December 2007. The rapid infusion rituximab protocol indicates to infuse rituximab at a rate of 150 mL/hr for 30 minutes then increase the rate to 275 mL/hr until the infusion is complete. This method is used to prevent rate calculation errors and ensures all infusions are infused over 90 minutes (±5 minutes) for doses between 500 mg and 1000 mg. Patients are premedicated with acetaminophen and an antihistamine (cetirizine or diphenhydramine). Additionally methylprednisolone, hydromorphone, diphenhydramine and epinephrine are available in the event of an infusion-related reaction. Patients are monitored for signs and symptoms of infusion-related reactions. If symptoms of a reaction occur, the infusion is stopped until symptoms resolve and is resumed at 50% of the previous rate and titrated every 30 minutes as tolerated. Patients must meet the following criteria in order to be eligible for rapid infusion rituximab:

- rituximab courses two to eight prescribed at standard doses given concurrently with chemotherapy,
- no grade 3/4 toxicity with previous course,
- subsequent rituximab monotherapy courses if no grade 3/4 toxicity with initial two courses,
- peripheral lymphocyte count < 4.8 k/uL,
- maintenance chemotherapy, following response to induction to chemotherapy.

This retrospective analysis included patients diagnosed with low-grade non-Hodgkin's lymphoma who received rapid infusion rituximab for maintenance therapy from December 2007 through November 2011. Patients were included if they were greater than 18 years of age, met eligibility requirements to receive rapid infusion rituximab per Moffitt protocol, and diagnosed with low-grade non-Hodgkin's lymphoma. Patients were excluded if they did not meet requirements for rapid infusion rituximab protocol. Data collection included diagnosis, treatment regimen prior to maintenance therapy, rituximab maintenance schedule, administration of premedications, and any adverse events. Adverse events were evaluated through documentation, vital signs, and administration of supportive care. The primary objective of this retrospective analysis was to evaluate the incidence of Grade 3 and Grade 4 toxicities with maintenance rapid infusion rituximab according to the Common Terminology Criteria for Adverse Events version 4. Secondary objectives included evaluating all infusion-related adverse events (Grades 1–4) with maintenance rituximab and evaluating correlation of adverse events with varying schedules of rituximab maintenance therapy. Descriptive statistics were utilized to analyze data.

## 3. Results

A total of 1,070 patients received rituximab as part of their treatment regimen from December 1, 2007 through November 25, 2011. Rapid rate maintenance rituximab therapy was given to 109 patients for a total of 647 doses. Approximately 54% of the patients were male. All of the patients received premedication with acetaminophen and an antihistamine as directed by the package insert. Patients with allergy or intolerance to diphenhydramine received cetirizine. Patients who had a prior infusion-related reaction with rituximab premedicated with corticosteroids in addition to acetaminophen and diphenhydramine. Each patient received an average of six doses of maintenance rituximab therapy at a rapid infusion rate, and the average infusion time for each dose administered was approximately ninety minutes. Administration of rituximab at a rapid rate saved approximately 68 minutes per patient (Table 1).

**Figure 1:**
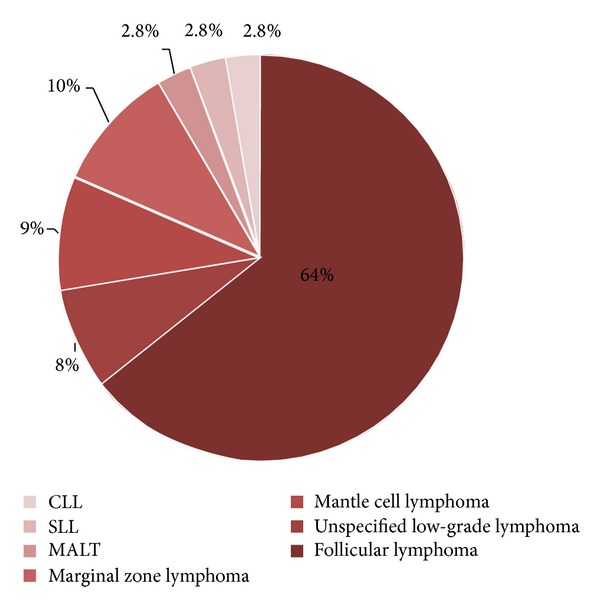
Diagnosis.

The majority of patients (64%) were diagnosed with follicular lymphoma and 8% had an unspecified low-grade lymphoma. The remainder of patients were diagnosed with one of the following low-grade lymphomas: mantle cell lymphoma, marginal zone lymphoma, mucosa-associated lymphoid tissue (MALT), small lymphocytic leukemia, and chronic lymphocytic leukemia (Figure 1).

**Figure 2:**
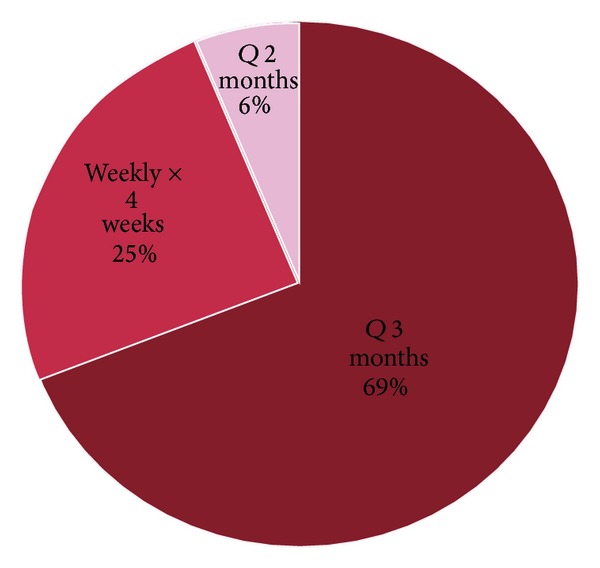
Rituximab Schedule.

Sixty-nine percent of patients received maintenance rituximab therapy every three months. Approximately 25% of the patients received rituximab weekly for four weeks every six months. A small minority of patients received rituximab maintenance regimen every two months based on data published from the PRIMA study [5] (Figure 2).

Out of the 109 patients analyzed, 3 patients developed an infusion-related reaction including one patient who developed a reaction twice.

All infusion-related symptoms resolved with administration of pharmacological management and/or supportive care. Two of the patients required pharmacological management with corticosteroids, additional antihistamines, and/or inhaled beta-agonists. Two of the patients received oxygen, and one patient received intravenous fluids. None of the patients required hospitalization due to the reactions.

## 4. Discussion

This analysis supports that rituximab can be safely administered as a rapid infusion during maintenance therapy. Our experience resulted in a low incidence of grade 3 reactions and no grade 4 reactions. The data also reveals the incidence of developing a grade 2 reaction is quite low. Out of the 109 patients analyzed, 3 patients developed an infusion-related reaction (Table 2). One patient experienced nausea (grade 1) with the first dose of the 3rd cycle of maintenance therapy. The other two patients experienced symptoms such as shortness of breath, facial flushing, and chest tightness (grade 3) (Table 3). All symptoms were alleviated with pharmacological therapy and/or supportive care. These patients were premedicated according to the package insert. None of these patients required hospitalization. Two of the patients were rechallenged at the rapid rate and tolerated therapy without further complications. In the patients who developed a grade 3 reaction, the incidence was too low to determine a correlation with the administration schedule of maintenance therapy.

When rituximab is administered at the standard rate, the patient's infusion time may last up to 6 hours. Longer administration time is inconvenient for the patient as well as nurses. With implementation of the rapid infusion method, the patient's infusion time has significantly decreased. As previously mentioned, H. Lee Moffitt Cancer Center administers rapid infusion rituximab at an initial rate of 150 mL/hr for 30 minutes followed by an increase of the rate to 275 mL/hr until completion of infusion. Our method of infusion varies from the package insert recommendation; however, the average infusion time was 90 minutes for our patients. With our method, over one hour of chair time was saved for each infusion. Rapid rituximab infusion improves the turn-around time for each patient and decreases resource utilization in the infusion center. 

With the low incidence of infusion-related reactions in combination with short administration time, rapid infusion rituximab is a feasible and attractive option for maintenance therapy. 

## Figures and Tables

**Table 1:** 

Patient demographics	*n* = 109

Male, *n* (%)	59 (54)
Median BSA (range), m^2^	2.05 (1.36–2.84)

Diagnosis	*n* (%)

Follicular lymphoma	70 (64)
Marginal zone lymphoma	11 (10)
Unspecified low-grade lymphoma	9 (8)
Mantle cell lymphoma	10 (9)
CLL/SLL	6 (5.5)
Mucosa-associated lymphoid tissue	3 (2.8)

Baseline laboratory parameters	k/μL (range)

Median lymphocyte count	1.24 (0.04–8.21)
Median WBC	5.38 (1.34–13.07)

Premedications	

Acetaminophen	99%
Diphenhydramine	75%
Cetirizine	23%
Corticosteroids	8.8%
Rituximab	
Median dose, mg	750 (410–1090)
Median no. doses	6 (1–20)
Mean infusion time, min	90 (85–97)

**Table 2:** Patient case.

Patient	1	2	3
Age (years)	46	51	66
Diagnosis	Low-grade follicular lymphoma	CLL/SLL	Marginal zone lymphoma
Treatment	CVP-R × 6 cycles	Weekly rituximab × 8 weeks	R-CHOP × 1 cycle followed by 4 weeks of weekly rituximab
Maintenance schedule	Q 3 months	Weekly × 4 weeks Q 6 months	Weekly × 4 weeks Q 6 months
Dose no. with reaction	5	Course 2, week 1	(a) Course 3, week 1 (b) Course 3, week 2
Grade of reaction	3	1	(a) 3 (b) 3
Rapid rate rechallenge	No	Yes	Yes

**Table 3:** Infusion-related reaction.

Rituximab-related reactions *n* = 109	
Grade 1	
Nausea Vomiting	1 (0.92%)
Grade 3	
Shortness of breath Throat swelling Facial flushing; chest tightness	3 (2.75%)

Total	4 (3.67%)
